# A Case of a Small-Breed Dog with Diet-Related Dilated Cardiomyopathy Showing Marked Improvements in Cardiac Morphology and Function after Dietary Modification

**DOI:** 10.3390/vetsci9110593

**Published:** 2022-10-27

**Authors:** Takahiro Saito, Ryohei Suzuki, Yunosuke Yuchi, Yuyo Yasumura, Takahiro Teshima, Hirotaka Matsumoto, Hidekazu Koyama

**Affiliations:** Laboratory of Veterinary Internal Medicine, School of Veterinary Science, Faculty of Veterinary Medicine, Nippon Veterinary and Life Science University, Tokyo 180-8602, Japan

**Keywords:** grain, myocardial function, strain, two-dimensional speckle-tracking echocardiography

## Abstract

**Simple Summary:**

Dilated cardiomyopathy is a cardiac disorder most commonly observed in specific dog breeds and is characterized by diffuse left ventricular systolic dysfunction and left ventricular enlargement. Recent studies have reported a potential connection between diet and dilated cardiomyopathy, and some studies have shown a positive effect of diet change on cardiac function and prognosis in dogs with diet-related dilated cardiomyopathy. However, these reports were from large-breed dogs and did not include detailed assessments of myocardial function, such as two-dimensional speckle-tracking echocardiography. We report an overview of our experience in a small-breed dog with a clinical diagnosis of dilated cardiomyopathy, in which dietary modification resulted in improved cardiac enlargement and myocardial dysfunction evaluated by two-dimensional speckle-tracking echocardiography. We suggest that it is necessary to suspect a dietary association with dilated cardiomyopathy, even in small-breed dogs. Furthermore, the prognosis for diet-related dilated cardiomyopathy in small-breed dogs may also be as good as in previous reports of large-breed dogs when changing to appropriate diets.

**Abstract:**

An 11-year-old intact female Papillion weighing 2.1 kg was referred to our institution with the main complaint of shallow, rapid breathing. At the first visit (day 0), although clinical signs improved due to the use of medication from the primary hospital, transthoracic radiography and echocardiography revealed left heart enlargement and left ventricular dysfunction. A clinical diagnosis of dilated cardiomyopathy (DCM) was made and oral administration of pimobendan, temocapril, and taurine was initiated. However, on day 10, the respiratory status worsened and furosemide was prescribed. On day 54, no significant improvement in heart size was observed. Additionally, the diet that this patient received met the recommendation for diet-related DCM by the U.S. Food and Drug Administration, and the patient’s diet was changed from a grain-free diet to a grain-containing diet. On day 1191, the patient’s respiratory status was stable and no clinical signs were observed. Transthoracic radiography and echocardiography revealed an improvement in left heart size. Additionally, improvements in the left and right ventricular myocardial strains were observed after changing the diet. We suggest that it may be necessary to suspect a dietary association with dilated cardiomyopathy, and a good prognosis might be expected by dietary modification, even in small-breed dogs.

## 1. Introduction

Dilated cardiomyopathy (DCM) is a cardiac disorder most commonly observed in specific dog breeds and is characterized by diffuse left ventricular (LV) systolic dysfunction and LV enlargement [1]. Genetic background is suspected to be the etiology of the disease, but other outbreaks due to nutrient deficiencies [2,3] and concurrent diseases, such as endocrinopathies [4], myocarditis [5], and chronic tachycardia [6], have also been reported. In 2018, the United States Food and Drug Administration released a report on the potential association between diet and DCM. Although a relationship between nutrients and cardiomyopathy has been reported, there have been recent reports of improved cardiac function in dogs with DCM due to dietary changes [7]. However, most of the dogs in these reports were large-breed dogs, and there are few reports on small-breed dogs [8], which account for a large proportion of dogs in Japan. We report an overview of our experience in a small-breed dog with DCM-like pathology, in which a change in diet significantly im-proved cardiac morphology and function.

## 2. Case Presentation

**Figure 1:**
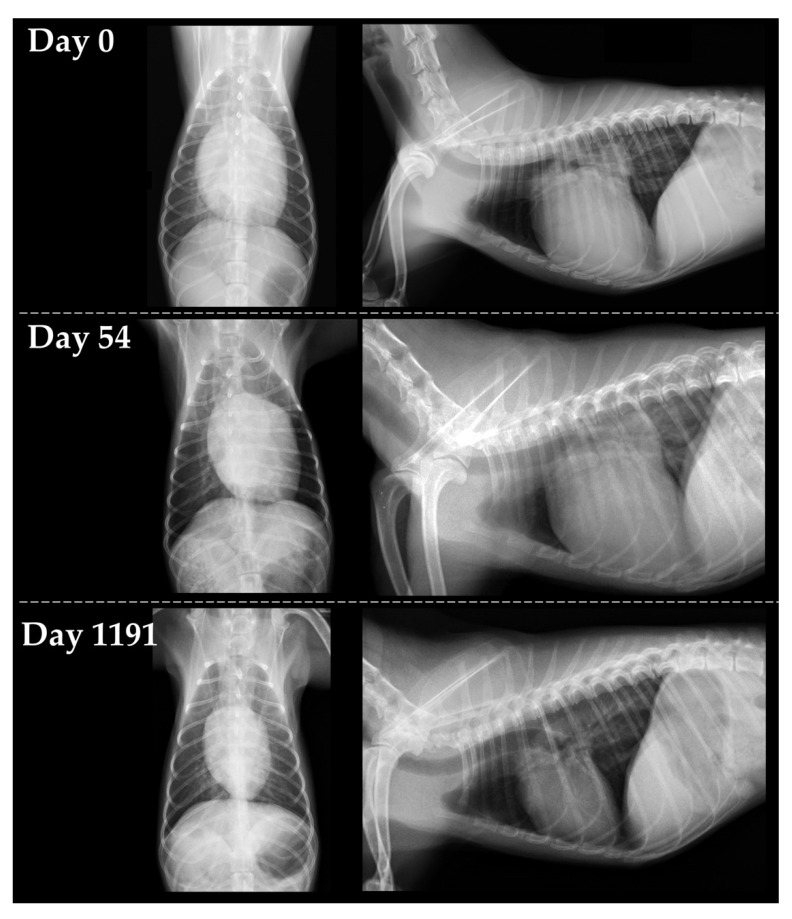
Changes in transthoracic radiography on day 0, day 54 and day 1191. Left figures represent the dorsoventral views, and right figures represent right lateral views.

**Figure 2:**
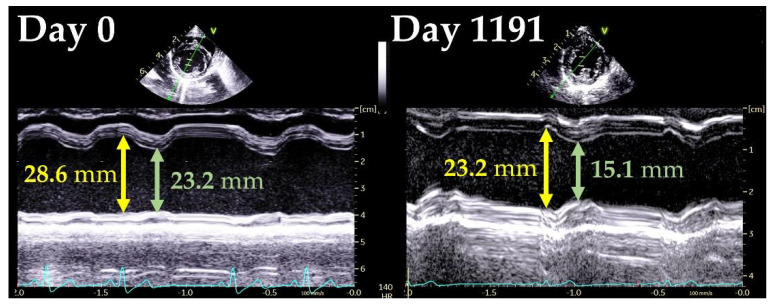
Changes in left ventricular internal diameter on day 0 and day 1191.

An 11-year-old intact female Papillion weighing 2.1 kg was examined in the primary hospital with the main complaint of shallow, rapid breathing. At that time, there was no heart murmur; however, a radiographic examination revealed an enlarged heart and an area of increased radiographic opacity in the posterior lobe of the right lung. Echocardiography revealed mitral regurgitation (MR). After treatment with furosemide (1.5 mg/kg), prednisolone (1.0 mg/kg), and enrofloxacin (5.0 mg/kg), dyspnea and increased lung field opacity on transthoracic radiography improved, but the patient came to our institution for a thorough examination. At the first visit (day 0), clinical signs improved and respiratory status was stable. Auscultation revealed a systolic murmur (Levine II/VI), but electrocardiography and noninvasive blood pressure measurements (systolic blood pressure, 139 mmHg) were normal. Transthoracic radiography revealed a slightly increased opacity in the lung field. The vertebral heart size (VHS) was 13.2 vertebrae, indicating cardiac enlargement (Figure 1). Conventional 2D and Doppler examinations were performed using Vivid 7 or Vivid E95 Ultra Edition echocardiographic systems (GE Healthcare) with a transducer of 3.5–6.9 MHz. The dog was manually restrained in the right and left lateral recumbent positions without sedation. The results of the echocardiographic measurement are summarized in Table 1. Echocardiography revealed MR and reduced regurgitation velocity, left atrial and LV enlargement, LV wall thinning, and reduced LV contractility. Transmitral early diastolic and late diastolic wave velocities (E and A, respectively) showed a restrictive pattern. The aortic blood velocity showed a high pre-ejection period to ejection time ratio (PEP/ET). Based on these findings, we suspected DCM and made a clinical diagnosis using a previously reported DCM diagnostic scoring system [1]. In this scoring system, a score of 3 points is given for matching one major criterion and 1 point for matching one minor criterion, with a total score of ≥6 points indicating a strong suspicion of DCM. The major criteria were defined as follows: systolic or diastolic LV dilatation, decreased sphericity index (≤1.65), and decreased fractional shortening (FS) (≤20–25%). The minor criteria were defined as follows: ventricular arrhythmia, atrial fibrillation, increased E-point-to-septal-separation (EPSS) (≥8.0 mm), increased PEP/ET (>0.4), mildly decreased FS (Figure 2), and enlargement of left atrial or biatrial enlargement. The LV end-diastolic volume index (EDVI) and LV end-systolic volume index (ESVI) measured by the modified Simpson method increased, indicating enlargement of the left ventricle. Furthermore, the ejection fraction (EF) decreased in the modified Simpson method. The LV sphericity index was low, indicating that the ventricle was more spherical than normal. The EPSS was within the reference range but close to the upper limit. Based on the above, three major criteria and two minor criteria were applicable in this case, with a total score of 11 points, suggesting a strong suspicion of DCM. Cardiac troponin I (cTnI) was measured and showed a high value of 1.105 ng/mL (normal range, 0.000–0.129 ng/mL). Therefore, a clinical diagnosis of DCM was made, and pimobendan (0.3 mg/kg BID), temocapril (0.1 mg/kg BID), and taurine (250 mg/head BID) were administered orally. However, on day 10, the respiratory status worsened and furosemide (2.0 mg/kg BID) was also prescribed.

On day 54, respiratory status improved, but radiographic examination in the primary hospital did not show a significant change in VHS (12.7 vertebrae). At that time, the diet 110 that the patient received met the criteria associated with diet-related DCM according to the U.S. Food and Drug Administration [7], and the patient’s diet was changed from a grain-free diet to a grain-containing diet. Ingredients in the product before the diet change mainly included: cured venison meat, venison meal, tapioca, peas, pea flour, lentils, chickpeas, canola oil, dried chicory root, dried rosemary, sodium chloride, potassium chloride, choline chloride, vitamins (vitamin A, vitamin D3, vitamin E, inositol, niacin, L Vitamin A, vitamin D3, vitamin E, inositol, niacin, L-ascorbic acid-2-polyphosphate, calcium d-pantothenate, thiamine nitrate, beta-carotene, riboflavin, pyridoxine hydrochloride, folic acid, biotin, and vitamin B12), minerals (zinc, copper, zinc oxide, manganese, copper sulfate, ferrous sulfate, calcium iodate, manganese oxide, and selenium yeast), DL-methionine, and L-lysine. On the other hand, the ingredients of the products after the diet change mainly included the following: rice, animal fats, corn, soy protein, beet pulp, soybean oil, wheat flour, vegetable fiber, fish oil, fructo-oligosaccharide, marigold extract, amino acids (DL-methionine, L-lysine, taurine, and L-carnitine), minerals (Ca, K, Cl, P, Na, Fe, Se, Zn, Mn, Mg, and I), vitamins (choline, vitamin E, vitamin C, Biotin, vitamin A, calcium pantothenate, niacin, vitamin B6, vitamin B12, vitamin B1, vitamin B2, vitamin D3, folic acid, and vitamin K3), preservative (potassium sorbate), and antioxidants (mixed tocopherol). No legumes were included in the products after the dietary change.

Subsequently, the patient was followed-up at the primary hospital. Approximately 3 years later, on day 1191, the patient was examined at our institution again. During this period, visits to our institution were difficult because the owner’s address was far away. The respiratory status was stable, and no clinical signs were observed. Additionally, there were no changes in oral medications (pimobendane [0.3 mg/kg BID], temocapril [0.1 mg/kg BID], taurine [250 mg/head BID] and furosemide [2.0 mg/kg BID]) since day 10. Physical examination, blood pressure measurements, and electrocardiogram did not show significant changes, but there was a marked improvement in the cTnI level to 0.003 ng/mL. On radiographic examination, there were no obvious abnormalities in the lung fields, and the VHS was markedly reduced to 9.2 vertebrae (Figure 1). Echocardiography revealed marked improvements in the LV and left atrial diameters and LV functional indicators (Table 1). The DCM diagnostic score for this patient did not meet any criteria, resulting in a total score of 0, which was below the diagnostic criteria for DCM. As improvement in cardiac function was observed after diet change, a clinical diagnosis of diet-related DCM was made, and the patient continued on a grain-containing diet. Regarding oral medications, furosemide and taurine were discontinued, while temocapril and pimobendan were continued in the same doses. The patient remained stable for 1262 days, with no recurrence of heart failure.

**Figure 3:**
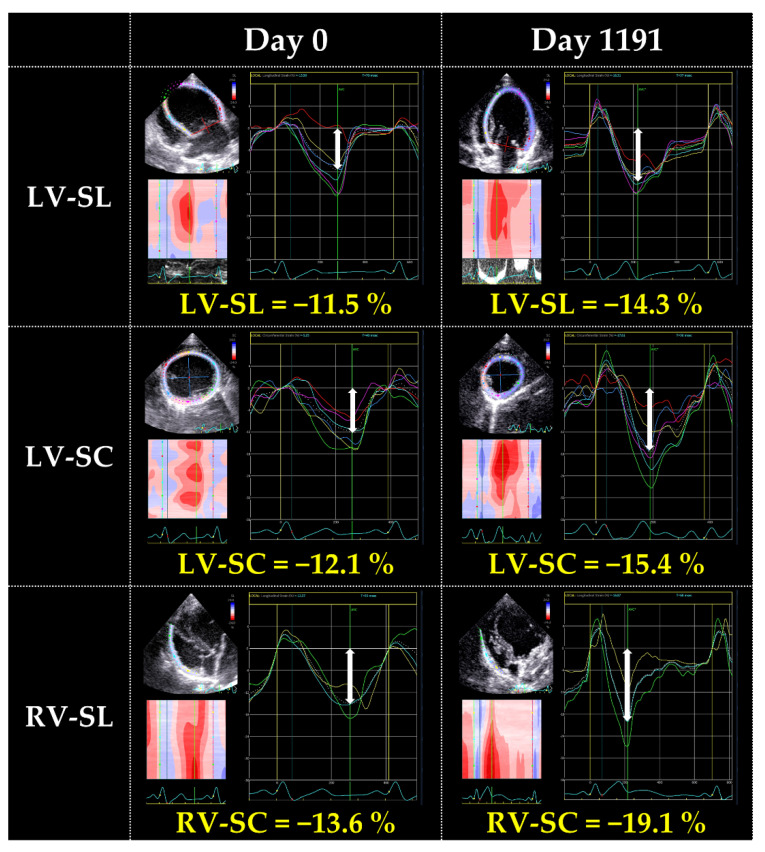
Changes in left ventricular longitudinal strain on day 0 and day 1191.

We then reviewed the case and performed myocardial motion analysis using two-dimensional speckle-tracking echocardiography (2D-STE). We measured the peak global strains in the longitudinal and circumferential directions (SL and SC, respectively) and the systolic strain rates in the longitudinal and circumferential directions (SrL and SrC, respectively). SL and SrL were measured in the LV and right ventricle (RV) using the left apical four-chamber view. SC and SrC were measured using the right parasternal short-axis view at the level of the papillary muscle. The mean values of the measurements for three consecutive cardiac cycles were used in these 2D-STE variables. We also measured torsion and torsion rate. For torsional deformations, a right parasternal short-axis view of the LV was used with recordings made at both the basal and apical imaging planes. For torsion results, representative values were used instead of continuous heartbeats. The observer variability of 2D-STE analysis in our laboratory has previously been described [9,10,11,12,13,14]. The reference range was based on previous reports [12,14,15,16,17,18,19,20,21,22]. On day 0, the SL and SC measured in the LV and RV were below the reported reference range for each of the parameters. SrL measured in the LV and RV was lower than each of the reference range. SrC was lower than the reference range. Torsion and torsion rate were lower than the reference range as well. On day 1191, these values showed improvement. In particular, substantial increases were observed in RV-SL, RV-SrL, torsion, and torsion rate (Table 2 and Figure 3).

## 3. Discussion

This patient was clinically diagnosed with DCM based on the clinical course and scoring system [1]. The patient was treated with recommended medication for DCM, such as pimobendan, angiotensin-converting enzyme inhibitor, and taurine; however, no significant changes in cardiac size were observed. In contrast, a change to a grain-containing diet resulted in significant improvement. These improvements were also evident in the analysis of conventional echocardiography and precise myocardial function assessed by 2D-STE [11,12,14]. Previous studies that analyzed the composition of grain-free diets showed low levels of taurine and B vitamins, which are components of carnitine, and high levels of compounds that inhibit carnitine metabolism. Peas were also mentioned as the materials most associated with their composition [23]. The diet that this patient had eaten until day 54 was a grain-free diet consisting mainly of venison and legumes such as peas. Therefore, it is possible that this case also had diet-related DCM, in which the grain-free diet induced a DCM-like pathology.

The extensive and multidirectional myocardial dysfunction was observed on day 0 through 2D-STE analysis. The 2D-STE variables in this case were worse compared with those in the previous study of Great Danes with idiopathic DCM [19], suggesting that the diet-related DCM may have the remarkable myocardial dysfunction. In this case, dietary modification caused improvement in myocardial dysfunction which did not improve with conventional medical therapy. In particular, torsional motion and RV myocardial deformation indicators, the precise myocardial performance indicator [9,10,15,24], showed the substantial improvement. These results suggest that assessment of myocardial function by 2D-STE may be useful to detect myocardial dysfunction induced by diet-related DCM, as demonstrated in hypertrophic and restrictive cardiomyopathy in cats [13,25,26,27]. However, 2D-STE could not identify the myocardial functional characteristics specific to the diet-related DCM to differentiate idiopathic DCM. Further studies that include a large number of dogs with DCM of various causes are expected in the future.

In previous reports, the most common breeds of dogs with DCM were large-breed dogs, such as Dobermans [28], but the dog in this case was a small-breed dog weighing 2.1 kg. Previous reports have stated that when DCM is diagnosed in atypical breeds, such as small breeds, it is reasonable to suspect a nutritional etiology, especially when dietary changes reversibly improve cardiac function [7]. Particularly in small breeds, there are few reports of heritability. Therefore, diet-related involvement should be adequately considered. In addition, current clinical diagnostic criteria do not include criteria related to dietary content. In this case, we were unable to differentiate sufficiently between the two until we confirmed the therapeutic response to the dietary changes. Therefore, the patient’s diet should be included in the diagnosis and should be confirmed by a thorough medical interview. Furthermore, previous studies have reported that dogs with DCM on grain-free diets have higher rates of congestive heart failure than those on grain-containing diets [7]. Dogs with DCM fed a grain-free diet, such as the dog in this case, should be considered at high risk for heart failure if diet modification is not made early enough and should be carefully monitored. Meanwhile, it has been reported that dogs with DCM with improved cardiac function after changing from a grain-free diet to an appropriate diet have a better prognosis than dogs with DCM despite being on a grain-containing diet [7]. In this case, a remarkable improvement in cardiac morphology and function was observed after changing to a grain-containing diet, and the dog is still alive with no recurrence of clinical signs 3 years and 5 months after the initial onset of heart failure. Overall, diet-related DCM, as in this case, can be expected to have a good long-term prognosis if managed with an appropriate diet.

There are limitations to this case report. The intervals between follow-ups have been significantly longer, and multiple medications have been administered over a period of approximately three years. These may have influenced the structural and functional changes observed after approximately three years.

## 4. Conclusions

This is one of the few reports of diet-related DCM in a small dog. The 2D-STE could detect the extensive and multidirectional myocardial dysfunction in dogs with diet-related DCM. As mentioned in the beginning of this paper, various factors contribute to the development of DCM. However, considering the recent diversification of dog diets, it may be necessary to suspect a dietary association among small-breed dogs. Furthermore, the prognosis for diet-related DCM in small-breed dogs may also be as good as in previous reports of large-breed dogs, as long-term maintenance of the disease was achieved through dietary modification in the present case.

## Figures and Tables

**Table 1:** Changes in conventional 2D and Doppler examinations on day 0 and day 1191.

Variables	Day 0	Day 1191
LVIDd (mm)	28.6	23.2
LVIDdN (cm/kg^0.294^)	2.3	1.8
LVIDs (mm)	23.2	15.1
LVIDsN (cm/kg^0.294^)	1.9	1.2
FS (%)	18.8	35.1
LA/Ao	2.4	1.3
E (m/s)	1.2	0.6
A (m/s)	0.3	0.9
E/A	4.1	0.7
PEP/ET	0.5	0.2
MR velocity (m/s)	4.6	5.9
EDVI (mL/m^2^)	108	36.6
ESVI (mL/m^2^)	75	19.6
Sphericity index	1.1	1.7
EPSS (mm)	0.7	0.3
EF (%)	30.5	46.3

LV, left ventricular; LVIDd, end-diastolic LV internal diameter; LVIDdN, LVIDd normalized by body weight; LVIDs, end-systolic LV internal diameter; LVIDsN, LVIDs normalized by body weight; FS, fractional shortening; LA/Ao, left atrial-to-aortic ratio; E/A, peak velocity of the early diastolic wave-to-peak velocity of the late diastolic wave ratio; PEP/ET, pre-ejection period to ejection time ratio; MR, mitral regurgitation; EDVI, LV end-diastolic volume index; ESVI, LV end-systolic volume index; EPSS, E-point-to-septal-separation; EF, ejection fraction.

**Table 2:** Changes in conventional 2D-STE examinations on day 0 and day 1191.

Variables	Day 0	Day 1191	Healthy Dogs [12,14,15]
LV-SL (%)	−11.6	−13.9	−14.9 ± 4.7
LV-SC (%)	−11.7	−13.1	−17.6 ± 2.5
RV-SL (%)	−13.5	−18.6	−26.9 ± 7.8
Systolic LV-SrL (s^−1^)	−1.1	−2.0	−1.9 ± 0.4
Systolic LV-SrC (s^−1^)	−0.9	−2.0	−2.1 ± 0.5
Systolic RV-SrL (s^−1^)	−1.2	−3.6	−4.2 ± 1.5
Systolic basal rotation (°)	−2.3	−3.7	−3.3 ± 3.4
Systolic basal rotation rate (°/s)	−55.6	−78.0	−119.9 ± 44.4
Systolic apical rotation (°)	4.8	10.7	12.9 ± 6.0
Systolic apical rotation rate (°/s)	65.7	89.8	181.4 ± 65.1
Systolic torsion (°)	5.1	11.7	13.6 ± 5.8
Systolic torsion rate (°/s)	74.6	136.8	154.7 ± 61.2

Data are expressed as mean ± standard deviation. LV, left ventricular; SL, peak global strain in the longitudinal direction; SC, peak global strain in the circumferential direction; RV, right ventricular; SrL, strain rate in the longitudinal direction; SrC, strain rate in the circumferential direction.

## Data Availability

The datasets used or analyzed during the current study are available from the corresponding author upon reasonable request.
